# Assessing parental stress and self-efficacy: A multisite feasibility study of parent-mediated physical activity interventions for children with developmental disabilities

**DOI:** 10.34172/hpp.43108

**Published:** 2024-12-30

**Authors:** Luis Columna, Scott McNamarra, Beth A Myers, Nienke Dosa, Ashlyn Barry, Kristi Roth, Christine E. Ashby, Byungmo Ku, Timothy Davis, Nikkia Borowski, Lisa M. Hooper

**Affiliations:** ^1^Department of Kinesiology, University of Wisconsin-Madison, Madison, Wisconsin, United States; ^2^Department of Kinesiology, University of New Hampshire, Durham, New Hampshire, United States; ^3^Inclusive Education, School of Education, Syracuse University, Syracuse, New York, United States; ^4^Department of Pediatrics, SUNY Upstate Medical University, Syracuse, New York, United States; ^5^School of Education, University of Wisconsin-Stevens Point, Stevens Point, Wisconsin, United States; ^6^Department of Adapted Physical Education, Yong-In University, Gyeonggi-do, South Korea; ^7^Department of Adapted Physical Education, State University of New York-Cortland, Cortland, New York, United States; ^8^Center for Educational Transformation, University of Northern Iowa, Cedar Falls, Iowa, New York, United States

**Keywords:** Behavioral changes, Developmental disabilities, Education, Nonprofessional, Health behavior, Online systems, Physical activity, Programmed instructions as topic, Teaching materials

## Abstract

**Background:**

Children with developmental disabilities often face barriers to engaging in physical activity (PA), impacting their health and quality of life. Parent-mediated interventions (PMIs) have shown promise to reduce these barriers, but little research explores online PMIs for parents of children with developmental disabilities. Thus, the purpose of this study was to assess the feasibility and impact of a multi-site collaborative online parent-mediated PA intervention on stress levels and self-efficacy among parents of children with developmental disabilities over a 12-week period.

**Methods:**

Participants (n=55) were parents of children with developmental disabilities, randomly assigned to intervention (n=27) or control (n=28) groups.

**Results:**

Recruitment rate was 58%, with an 80% retention rate. The feasibility of online delivery was demonstrated, allowing participation from various locations. An analyses of covariance (ANCOVA) with parental sex and education level as covariates revealed no significant interaction effect between group and time for parenting self-efficacy score (PSE), F(1, 104)=0.118, *P*=0.732, or PSI, F(1, 104)=0.196, *P*=0.659. The mean PSI difference (pre-post) was -0.38 (CI: -10.57 to 9.80) for the experimental group and 2.64 (CI: -9.61 to 14.91) for the control group, while the mean PSE difference was -4.41 (CI: -29.33 to 20.49) and 4.75 (CI: -23.22 to 32.73), respectively.

**Conclusion:**

Future research should explore the integration of hybrid PMIs in conjunction with qualitative measures to facilitate a deeper understanding of the multifaceted factors influencing parental engagement in PA interventions for children with developmental disabilities.

## Introduction

 Developmental disabilities encompass a range of conditions involving functional limitations across physical, cognitive, and behavioral domains.^1^ These disabilities often result in significant challenges with gross and fine motor skills due to underlying neurological impairments,^2-5^ contributing to lower physical activity (PA) engagement compared to peers without disabilities.^6,7^ Beyond neurological deficits, societal barriers, such as a shortage of trained professionals, inaccessible programs, and financial constraints, further complicate access to PA for children with developmental disabilities.^8–10^ Consequently, these children face increased risks of secondary health conditions like obesity and diabetes, which significantly impact their quality of life.^11^

 These barriers are compounded by personal factors, including parents’ safety concerns, lack of time, and inadequate support systems, which further restrict their children’s PA participation.^12,13^ Given their essential role in their children’s lives, parents are uniquely positioned to mitigate these barriers. Parental attitudes, knowledge, and support significantly influence children’s PA engagement.^14-17^ For instance, Ku et al^17^ found that parental behaviors, such as encouraging PA and modeling active lifestyles, predict PA levels in children with developmental disabilities. A key factor in these behaviors is parental self-efficacy, which refers to a parent’s confidence in their ability to facilitate PA opportunities and is essential for fostering consistent PA participation.^14^ Research indicates that enhancing self-efficacy through skill-based training can empower parents to overcome barriers and support their children more effectively.^15,16^

###  Parental stress and self-efficacy

 Raising a child with a developmental disability is associated with heightened parental stress, often due to the diverse abilities and unique needs of their children.^18,19^ This stress is further compounded when parents experience low self-efficacy, which limits their ability to facilitate PA opportunities and creates a cycle that hinders participation.^12,13^ Providing parents with practical strategies and skills to promote PA can help alleviate stress and enhance self-efficacy, ultimately benefiting both parents and children.^15,17^

 Parent-mediated interventions (PMIs) have demonstrated positive outcomes by equipping parents with the skills to support their children’s PA. For example, Prieto et al^17^ and Columna et al^15^ implemented PMIs where parents practiced PA facilitation at home, supported by professionals with expertise in motor skills and sensory activities. These interventions were delivered both in-person and online, with parents reporting benefits such as flexibility and hands-on guidance. However, while online formats address logistical barriers, existing studies have yet to fully explore their impact on parental stress and self-efficacy.^20-22^

 This study builds on prior research by assessing the feasibility and impact of a multi-site, 12-week online PMI designed to reduce parental stress and enhance self-efficacy in supporting their children’s PA.

###  Research questions

1. To what extent is a multi-site collaborative online parent-mediated PA intervention feasible in terms of recruitment and retention?
2. How does this 12-week online parent-mediated PA intervention impact stress in parents of children with developmental disabilities?
3. How does this 12-week online parent-mediated PA intervention impact self-efficacy levels in parents of children with developmental disabilities?

## Material and Methods

###  Research design

 A priori power analysis was performed to determine the necessary sample size for our study utilizing a repeated measures multivariate analysis of variance (MANOVA). The analysis was conducted to achieve a power of 0.90 for detecting a medium effect size (f(V) = 0.5) with an alpha level of 0.05, considering the within-between interaction of our two intervention groups across two measurement times. The power analysis indicated that a total sample size of 45 participants was required to adequately power the study. This sample size calculation ensures a high likelihood (actual power ≈ 0.906) of detecting statistically significant effects, thus substantiating the robustness and reliability of the anticipated findings.

 We conducted a parallel-group randomized control trial, employing equal randomization (1:1) to assign parents of children with developmental disabilities into one of two conditions: the workshop (intervention) group (n = 35) or the control group (n = 35). While the control group did not participate in the 12-week online parent-mediated PA intervention, they received all materials and equipment upon its completion.

###  Participants

 A convenience sampling technique was used to recruit parents of children with developmental disabilities from Iowa, New York, and Wisconsin. Although convenience sampling was used to recruit participants, randomization was applied once the sample was formed to ensure equal group allocation, making this a randomized control trial in terms of intervention assignment. To ensure balanced representation across states, a stratified randomization approach was employed. Participants were first divided into strata based on their state of residence (Iowa, New York, Wisconsin). Within each stratum, random allocation to either the control or experimental group was conducted using Microsoft Excel. Random numbers were generated using the RAND() function in Excel, and participants within each state were sorted based on these random numbers. Subsequently, participants were alternately assigned to the control or experimental group to ensure an even distribution within each state. Finally, groups were combined across states to form the intervention and control groups, maintaining balance and enhancing the study’s internal validity.

 We confirm that the procedures followed in this study adhered to the ethical standards outlined in the Helsinki Declaration of 1975. Following Institutional Review Board approval, participant recruitment involved reaching out through various channels, such as the researchers’ social media (e.g., Facebook). In addition, local disability organizations sent participant recruitment information through their listservs. Subsequently, a snowball sampling technique was used to identify future participants, utilizing the research team’s professional networks.^23^ Although convenience and snowball sampling could lead to bias in selecting participants,^24^ the random assignment to groups helped reduce these concerns and improved the study’s internal validity.^25^ The inclusion criteria for parents in the study were (*a*) identifying as the primary guardian of a child with a developmental disabilities diagnosis between the ages of 4 and 11 years, (*b*) having the ability to understand and communicate in English (both spoken and written), and (*c*) being willing to be a participant in the intervention. Even though we did not collect data on the children, it was required that the children had a diagnosis of developmental disabilities, were between the ages of 4 and 11 years, and could walk independently (See Table 1).

###  Intervention 

 The 12-week intervention was adapted from an in-person program and consisted of workshops covering the following topics: (a) sensory motor activities, (b) communication, (c) physical activity (PA), and (d) sports.^15,17^ The online workshops were conducted at three-week intervals, aiming to teach parents of children with developmental disabilities strategies to improve their child’s PA participation. During the workshops, informational sessions were presented by professionals (e.g., adapted physical education, special education practitioners and researchers) who were specialists in the target topic for each workshop, with extensive experience in these areas. These professionals provided either live demonstrations or used videos to demonstrate techniques, giving parents a clear understanding of how to apply the strategies with their children. Following the workshops, parents engaged in question-and-answer sessions with the experts. In addition to the core topics, each workshop incorporated essential content and techniques for PA and fundamental motor skills to equip parents with the necessary skills to teach their children.Towards the end of the workshops, parents were provided with an opportunity to practice what they learned with their child and discuss successful strategies with the experts and other parents. The workshops, which were conducted via Zoom, had a duration of approximately 3 hours each.

 Parents were encouraged to apply the skills taught in each workshop by engaging in PA, with the goal of aligning with the CDC’s Physical Activity Guidelines, which recommend 60 minutes of daily activity for children and youth.^26^ During the workshops, parents were taught about PA guidelines and encouraged to incorporate PA into their routines, with the goal of reaching 60 minutes a day, though it was acknowledged that this might not be feasible for all families every day. In addition, parents were provided with supplemental materials to assist their PA engagement, including a mobile application (app) and equipment. The mobile app included a collection of more than 200 interactive games, each equipped with mechanisms to adjust the level of difficulty, allowing for activities tailored to the needs of each participant. To ensure parents could effectively engage with the material, before each workshop, parents were given access to the games relevant to that session’s content through the app. They were encouraged to use these games with their children and had continued access to the games from previous workshops throughout the intervention. Additionally, the study team provided technical assistance (e.g., tips on how to download the app) via email, text message, and telephone between workshops. Moreover motivational text messages were sent on alternating dates three times per week to encourage participation with the equipment and to remind them about the next workshops. Families involved in the intervention were provided with all the necessary equipment (e.g., balls, cones) to actively participate in the games and activities within their own homes between each workshop. The control group received instructions to maintain their regular routines and engage in their usual activities throughout the intervention period.

###  Trial feasibility

 The primary outcomes for assessing trial feasibility included: (*a*) recruitment and retention and (*b*) parental stress and parental self-efficacy data collection.

####  Recruitment and retention

 The calculation of the recruitment rate involved dividing the number of eligible participants who initiated the intervention by the number of participants assessed for eligibility. A recruitment rate of at least 50% was deemed feasible, considering its comparability to a previous PMI for children with developmental disabilities.^27^ Retention rate, defined as the proportion of individuals who completed the study until the post-test out of the total number assessed for eligibility, was also considered. Comparable parent-mediated feasibility studies have reported retention rates ranging from 76% to 95%.^27,28^ An acceptable measure of trial feasibility was considered to be a retention rate of at least 76%.

###  Data collection

 Prospective families completed an initial online questionnaire to confirm eligibility. This questionnaire gathered information such as child’s age and disability diagnoses. Upon receipt of the questionnaire, eligible participants were contacted to provide detailed study information and obtain consent. Before and after the intervention, participants completed two questionnaires: one assessing parental stress and the second measuring parental self-efficacy. Details about these instruments will be provided below. Both intervention and control group parents completed these questionnaires, with only one caregiver per child per family invited to participate.

####  Demographic data sheet

 For this study, demographic information on both the parent and the child was collected using a comprehensive data sheet. The sheet captured various demographic variables including age, gender, socioeconomic status, the child’s age, ethnicity, state of residence, educational background, employment status, and marital status among others.

####  Parental stress

 The Parenting Stress Index-short form (PSI)^29^; was designed to assess assess parent’s perceptions of difficulties and feelings associated with the demands of parenting.^30^ This questionnaire consists of 36 Likert scale items (1 = *Strongly Disagree*, 5 = *Strongly Agree*). Higher scores are indicative of greater levels of parenting stress. The measure has three sub-scales: (1) Parental Distress refers to the degree of distress experienced by parents because of personal issues; (2) Parent-Child Dysfunctional Interaction pertains to the way a child fulfills the expectations set by their parent; and (3) The Difficult Child Assessment Tool evaluates the behavioral attributes of a child that contribute to their level of manageability, distinguishing between those that make them easy or difficult to handle. The PSI has established reliability and validity.^29^ In a previous study, the reliability of the overall PSI-SF was α = 0.91.^20^ In the present study, the internal consistency reliability of the items was α = 0.92 (total stress).

####  Parenting self-efficacy

 The Tool to Measure Parenting Self-Efficacy (TOPSE)^31^; was designed to assesses the multi-dimensionality of parent self-efficacy. Based on the principles of self-efficacy theory,^32^ the TOPSE incorporates the perspectives and experiences of parents from diverse cultural, educational, and social backgrounds.^31-33^ The TOPSE consists of 48 statements within eight scales, each subscale is composed of six statements and represents a distinct dimension of parenting: emotion and affection, play and enjoyment, empathy and understanding, control, discipline and boundaries, pressures, self-acceptance, learning and knowledge. The items are rated on an 11-point Likert scale (0 = *Completely Disagree*, 10 = *Completely Agree*). The scale contains positively and negatively worded items, and the responses are summed to create a total score; lower scores indicate lower levels of parenting self-efficacy. Previous studies have provided support for the reliability and validity of TOPSE.^33^ For instance, in a previous study, the reliability of the overall TOPSE was α = 0.89. In the present study, the internal consistency reliability of the items was α = 0.92 (total self-efficacy).

###  Data analysis

 Descriptive analysis (e.g., mean, frequency) was conducted for demographic information of participants and scores of PSI and TOPSE. For the primary analysis, only participants who completed both the pre- and post-test were considered (n = 55, retention rate was 80%). To address these missing values, the Little’s test for Missing Completely at Random (LMCR) analysis was conducted. The outcome of the LMCR indicated non-significance, χ2 = 165.35, df = 309, *P* = 0.98, suggesting the absence of data in the survey was likely due to random factors rather than any systematic bias. Consequently, missing values were inputted using the Expectation–Maximization algorithm.

 Levene’s test for equality of error variances was conducted to assess the assumption of homogeneity of variances. The results indicated that the variances of the PSI and TOPSE were not significantly different across the groups, F(3, 106) = 1.155, *P* = 0.331 and F(3,106) = 1.45, *P* = 0.231, respectively. Additionally, in examining the normality of the data, Shapiro-Wilk tests were utilized. For the variable PSI and TOPSE, it indicated that the data were normally distributed, W = 0.989, *P* = 0.488, W = 0.977, *P* = 0.06, respectively. Based on these findings, parametric tests were deemed appropriate for the main analysis.

 To assess the impact of the PA intervention on parental stress and self-efficacy, two sets of analyses were conducted. Firstly, two separate 2 × 2 repeated measures ANOVA tests were performed with time (pre and post) and group (control and experimental) as the factors. This allowed for an examination of the main effects and interaction between time and group on both parental stress and parental SE.

 Secondly, to account for potential confounding factors, two separate analyses of covariance (ANCOVA) were carried out. These analyses aimed to evaluate the influence of covariates—child’s sex and parental educational level—on the outcomes of parental stress and parental SE. By including these covariates, we sought to control for their potential effects and isolate the specific impact of the intervention on the measured outcomes. To measure the effect size between the pre and post conditions within each group, Cohen’s *d* equation was employed. Effect sizes are considered small (*d* = 0.2), medium (*d* = 0.5), or large (*d* = 0.8). All statistical analyses were conducted using IBM SPSS Staistics (version 28.0. Armonk, NY: IBM Corp).

## Results

**Figure 1:**
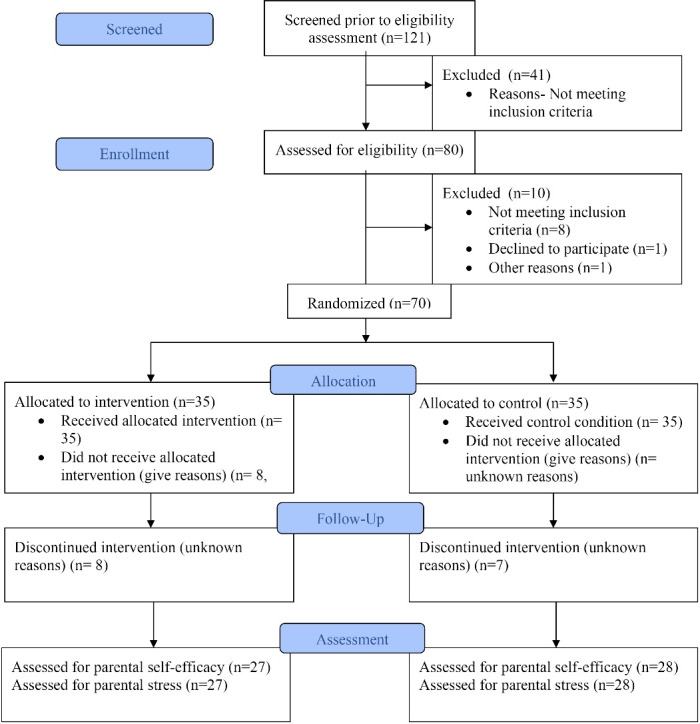
CONSORT flow diagram of participant enrollment, allocation, follow-up, and assessment for the intervention and control groups

 We recruited 121 potential families with children diagnosed with developmental disabilities, covering one Eastern and two Western regions of the United States. Initial screening questionnaires were completed by 121 families. Among them, 41 were excluded due to failure to meet eligibility criteria (e.g., child’s age). Subsequently, 80 potential participants underwent eligibility assessments, leading to exclusions based on non-compliance with inclusion criteria (*n* = 8), refusal to participate (*n* = 1), or unspecified reasons (*n* = 1). The remaining 70 families were stratified by age, gender, and geographical location of the child and randomly assigned to either the intervention (*n* = 35) or control group (*n* = 35). The randomization procedure was implemented using computer-generated random numbers (see Figure 1).

###  Recruitment and retention 

 In this study, out of the 121 families initially recruited, 70 successfully completed the eligibility criteria and were initially enrolled in the study, resulting in a recruitment rate of 58%. Among these 70 families, 55 completed the intervention, with 27 in the intervention group and 28 in the control group. Among the 15 families who did not complete the study, 5 completed the pre-test but did not follow up to continue, while the others either started or did not complete the pre-test. Notably, the study achieved a retention rate of 80%, as all 55 families who completed the intervention also completed the study. All 27 families in the intervention group confirmed receiving the equipment before the workshop, whereas families in the control group obtained their equipment upon concluding the study.

###  Intervention safety and receiving of the equipment

 Participants in both conditions confirmed receiving all the equipment. Specifically, the intervention group received the equipment two to three days before each workshop, totaling four boxes of equipment.

###  Parental stress and parental self-efficacy scores

Table 2 includes the mean scores of PSI and TOPSE in each group. The 2x2 repeated measures ANOVA did not reveal a significant time-main effect on PSI, F(1, 108) = 0.03, *p* = 0.86. However, there was a group-main effect on PSI, F(1, 108) = 9.22, *p* = 0.003, indicating a significant difference in the PSI scores between the control group and the intervention group. The analysis showed no significant interaction effect on PSI, F(1, 106) = 0.12, *p* = 0.73. Effect sizes for PSI from the pre to post intervention in the experimental and control groups, calculated using Cohen’s *d*, were 0.03 and 0.09, respectively.

**Figure 2:**
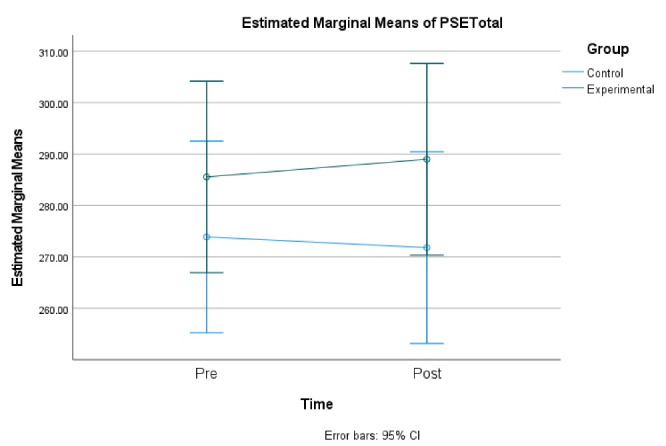
Pre-post Mean Parental SE Scores by Group

 In a parallel analysis, another 2x2 repeated measures ANOVA did not indicate a significant time-main effect on TOPSE, F(1, 108) = 0.005, *P* = 0.94. Similarly, there was no significant group-main effect on TOPSE, F(1, 108) = 2.36, *P* = 0.13, and no interaction effect on TOPSE, F(1, 106) = 0.09, *P* = 0.77. The effect sizes for TOPSE from the pre to post intervention in the experimental and control groups, computed using Cohen’s *d*, were 0.35 and 0.23, respectively (See Figure 2).

 An ANCOVA was conducted to examine the impact of group and time on PSI, with child’s sex and parental education level serving as covariates. Among the covariates, neither sex, F(1, 104) = 0.141, *P* = 0.708, nor education level, F(1, 104) = 0.856, p = 0.357, significantly contributed to the model. The main effect of group was statistically significant, F(1, 104) = 8.862, *P* = 0.004, suggesting that group differences significantly predicted PSI. The effect of time, F(1, 104) = 0.090, *P* = 0.765, as well as the interaction between group and time, F(1, 104) = 0.118, *P*= 0.732, were not significant, indicating that PSI did not change significantly over time and the effect of group on PSI did not vary across different time points.

 Another ANCOVA was conducted for TOPSE, which revealed that none of the covariates or predictors reached statistical significance. Specifically, child’s sex did not significantly predict TOPSE, F(1, 104) = 0.017, *P* = 0.896. Similarly, parental educational level did not significantly influence PSE Total scores, F(1, 104) = 1.174, *P* = 0.281. Regarding the predictors, the group also did not reach statistical significance, F(1, 104) = 2.317, *P* = 0.131, implying that group alone was not a significant determinant of TOPSE. The factor of time showed no effect, F(1, 104) = 0.002, *P* = 0.964. Lastly, the interaction between group and time was not significant, F(1, 104) = 0.196, *P* = 0.659.

**Table 1:** Demographic information of each group

	**Experimental group**	**Control group**	***P***
States			0.35
Iowa	4	2	
New York	13	16	
Wisconsin	10	10	
Parent’s sex			0.91
Male	2	2	
Female	25	26	
Parental race			0.41
White	22	22	
Black or African American	2	3	
Hispanic or Latino	3	3	
Marital status			0.70
Married/living with partner	21	23	
Divorced	3	1	
Never married	3	4	
Household Income			0.72
under $10,000	1	1	
$10,000-$24,999	1	2	
$25,000-$44,999	4	1	
$45,000-$74,999	6	7	
$75,000-$99,999	7	8	
Over $100,000	8	9	
Child age			0.55
0-5 years	9	8	
6-12 years	18	20	
Child sex			0.19
Boys	19	23	
Girls	8	5	

Note. *P* = *P *values of chi-square tests

**Table 2:** The descriptive results and the main effects of groups (experimental and control) and time (pre and post intervention) on PSI and PSE scores in parents of children with developmental disabilities

**Measure**	**Groups**	**Pre** **(Mean; SD)**	**Post** **(Mean; SD)**	**Group effect (F, ** ***p*** **)**	**Time effect (F, ** ***p*** **)**	Group*Time effect (F, P)
PSI	Experimental (n = 27, 27)	118.98 (18.28)	119.36 (19.36)	9.22, 0.003	0.03, 0.86	0.12, 0.73
	Control (n = 28, 28)	108.57(23.81)	105.92 (21.39)			
TOPSE	Experimental (n = 27, 27)	285.55 (45.40)	289.97 (46.67)	2.36, 0.13	.0054,.94	0.09, 0.77
	Control (n = 28, 28)	276.56 (53.70)	271.80 (49.56)			

Note. PSI = parenting stress index scores, PSE = parenting self-efficacy scores.

## Discussion

 The purpose of this study was to assess the feasibility and impact of a multi-site collaborative online parent-mediated PA intervention on stress levels and self-efficacy among parents of children with developmental disabilities over a 12-week period. The results of this study indicated that it is feasible to recruit and retain parents for a multi-site collaborative online parent-mediated PA intervention. Although the intervention demonstrated promise in terms of recruitment and retention, its impact on parental stress levels and self-efficacy was less clear. No significant differences were observed between the intervention and control groups regarding these two variables, but the small effect size improvements suggest that the intervention may still hold potential for enhancing parental self-efficacy.

###  Recruitment and retention

 The recruitment and retention rates documented in this study align with those observed in previous research involving children with developmental disabilities.^15,27,28^ Parents of children with developmental disabilities often face numerous caregiving responsibilities, posing challenges in prioritizing PA.^12,34^ We successfully recruited 58% of eligible participants, employing effective strategies such as sending motivational text messages and providing access to a mobile application containing PA strategies and resources. During workshops, we utilized common teaching strategies from physical education, such as visual schedules and cues, to enhance participant engagement. A total of 55 families, with 27 in the intervention group and 28 in the control group, completed the study, resulting in an 80% retention rate. While our retention rates align with those reported by Columna et al,^15^ Novak-Pavlic et al,^34^ and Matheson et al^28^ our recruitment rates surpassed the 50% reported by them, maybe due to our larger sample size.

 This study represents one of the first investigations in which parents of children with developmental disabilities actively participated in a multisite online PA intervention aimed at reducing parental stress and increasing parental self-efficacy. The primary objective of this study was to evaluate the feasibility of recruiting families from various states, exploring the potential to extend the reach and impact of evidence-based interventions to a broader participant base. Furthermore, a key lesson learned during the recruitment process emphasizes the importance of researchers earning the trust of participants and establishing connections with organizations providing services to these families. In addition, maintaining constant communication among leaders at each site proved to be essential.

###  Parental stress

 Stress among parents is multifaceted, often connected to various extrinsic factors such as child characteristics and the parent’s own resilience.^35,36^ Parents of children with developmental disabilities frequently face unique challenges and stressors related to raising a child with a disability, as well as societal pressures that can negatively affect stress levels.^37,38^ This complexity should be considered when evaluating the impact of online PMIs on parental stress levels.

 Existing literature has established positive associations between parental stress and their engagement in PMIs.^39^ Prior studies have shown that low-intensity PMIs can reduce stress.^39,40^ The current intervention was designed to be supportive, with simple and manageable learning tasks aimed at reducing stress by keeping learning activities incremental. In this study, there were no significant changes in parental stress levels, which may be due to the balanced intensity of the intervention.^41^ Additionally, maintaining stable stress levels could facilitate greater parent engagement in activities outside of the intervention and support the integration of these activities into their daily routines.^20,21^

 However, the online nature of the intervention may have limited opportunities for real-time support, which is crucial for alleviating stress during moments of uncertainty.^42^ While parents had access to resources and guidance, the absence of immediate support during independent practice may have hindered their ability to manage stress effectively. Future studies should consider incorporating real-time support or personalized check-ins to better address parental stress during intervention activities.^43^

###  Parental self-efficacy 

 The intervention demonstrated some promising potential for improving parental self-efficacy, as small effect size increases were observed among participants in the intervention group. While these improvements were not statistically significant, they suggest that the program may have had a subtle positive influence on parents’ confidence in supporting their children’s PA. Even small gains in self-efficacy can be meaningful, given the important role parents play in promoting PA and motor skill acquisition for their children with developmental disabilities.^14,44^

 One possible explanation for the lack of significant improvement may be related to the challenges associated with enhancing self-efficacy through online interventions.^45^ Bandura’s self-efficacy theory emphasizes the importance of mastery experiences, vicarious experiences (watching others perform the skill) and feedback (verbal persuasion) to enhance self-efficacy.^32^ Although the intervention provided instruction and opportunities for parents to ask questions in a supportive environment, the lack of immediate feedback during independent practice may have limited further gains in self-efficacy.^46,47^ While the flexibility of the intervention was appreciated by families, the absence of real-time support during practice may have reduced the program’s overall impact.

 Given the pilot nature of this study, we did not collect detailed data on how frequently parents applied the skills they learned or practiced with the app.^48,49^ Nonetheless, the modest improvements observed in this study suggest that with further refinement, such interventions hold great promise for boosting parental self-efficacy and the PA participation of children with developmental disabilities. Further discussion on potential improvements is addressed in the future studies section.

###  Implications for future research

 Although the findings from this study should be viewed as preliminary, the study and supporting literature pointing to various accessibility issues for parents of children with developmental disabilities in accessing quality PMIs emphasize the need to explore alternative delivery methods for PA interventions. A potential pathway for future research is the development of hybrid PMIs that combine online and in-person components to maximize effectiveness and participant participation. Future research could enhance these interventions by integrating more interactive features, such as real-time check-ins, live coaching, or synchronous sessions, to provide immediate feedback and reinforce mastery experiences. This could address the limitations observed in the current study related to parental stress and self-efficacy.

 Additionally, incorporating qualitative measures will provide richer insights into the experiences and perspectives of both parents and children with developmental disabilities, enhancing our understanding of the intervention’s impact. Qualitative research could explore how the intervention influences not only stress and self-efficacy but also other aspects of parental involvement, such as motivation, barriers, and long-term engagement in PA. Qualitative feedback from participants could also reveal subtle changes in family dynamics and provide direction for tailoring interventions to meet the specific needs of diverse families.

 Additionally, exploring the optimal level of intervention intensity—neither overwhelming nor too low—will be important to ensuring the program effectively supports parents while managing stress. The modest improvements observed in parental self-efficacy suggest that, with further refinement, these interventions hold great promise for improving PA participation among children with developmental disabilities.

 Finally, the nature of this pilot study limited our ability to ensure that parents actively practiced the skills learned to increase their children’s PA. Collecting data on the time parents spend using the app and practicing these skills will provide information into engagement levels and their impact on outcomes. Similarly, future research should collect child outcomes (e.g., fundamental motor skills) to offer a more holistic perspective on the intervention’s impact.

## Limitations

 The limitations of this study should be acknowledged. First, this study had a small sample size with some levels of attrition. Future studies should seek to gather a larger sample and identify incentives for retention (e.g., monetary compensation, PA equipment), as this could yield more reliable and comprehensive insights into the effects of the intervention. Second, the presence of differences in pre-test stress levels within the intervention and control groups should be noted. Specifically, parents in the intervention group had higher stress levels at pre-test. Thus, it is reasonable to assume that these differences might have influenced the outcomes and should be considered when interpreting the results related to parenting stress. Future research should aim to minimize such discrepancies at the study onset. Last, while we randomized families based on the gender and age of their children, we did not randomize based on stress levels and self-efficacy. Future studies should consider incorporating these factors into their randomization process. Additionally, while our app provided access to games and activities, it did not track the amount of time parents spent using the app, practicing what was presented in the workshops, or using the equipment with their child. Future studies should consider using apps that can measure and capture these variables to better understand engagement levels and their impact on outcomes.

## Conclusion

 This study explores the feasibility and impact of a 12-week online PMI for parents of children with developmental disabilities. Although statistically non-significant, the study reveals an increase in parental self-efficacy among the intervention group, suggesting that online PMIs could hold promise in real-world settings. While the findings suggest that online PMIs may provide useful support for parents in facilitating their children’s PA, they also highlight the unique challenges faced by families with children with developmental disabilities. Despite acknowledged limitations, including a small sample size and statistically non-significant results, this study contributes to advancing interventions by recognizing the successes and challenges in recruitment and retention. Developing online PA interventions that incorporate more interactive and supportive measures could improve their impact on parental stress and self-efficacy. The findings from this study set the stage for future research to optimize support programs for parents and children with developmental disabilities.

## Competing Interests

 None.

## Ethical Approval

 Human subjects approval was obtained from the University of Wisconsin-Madison Education and Social/Behavioral Sciences Institutional Review Board (IRB; protocol number 2022-0165). All participants provided written informed consent to participate.
